# Recent Advances in Ablative Therapies for Hepatocellular Carcinoma

**DOI:** 10.3390/cancers17193251

**Published:** 2025-10-07

**Authors:** Saad Abu Zahra, Arsalan Nadeem, Ashima Kundu, Nick Gibson, Ali Haggaz, Kent T. Sato, Robert J. Lewandowski, Andrew C. Gordon

**Affiliations:** Department of Interventional Radiology, Northwestern University, Chicago, IL 60611, USA

**Keywords:** hepatocellular carcinoma, image-guided ablation, locoregional therapy

## Abstract

**Simple Summary:**

Liver cancer is often diagnosed at a stage when surgery is not possible. In these cases, ablation therapies can destroy tumors directly in the liver using heat, cold, electricity, or sound waves. This review explains recent advances in these treatments and compares their benefits and limits, with the goal of guiding future care and research.

**Abstract:**

Hepatocellular carcinoma (HCC) is a leading cause of cancer-related mortality worldwide, with curative surgical interventions feasible for a minority of patients. This review highlights recent advances in thermal (e.g., radiofrequency ablation, microwave ablation, and cryoablation) and nonthermal (e.g., ethanol ablation and irreversible electroporation) ablative modalities as curative-intent alternatives to surgery. Evolving applications of transcatheter intra-arterial radioembolization (TARE) with ablative dosimetry will be explored, and histotripsy, an emerging technology, will be introduced.

## 1. Introduction

Hepatocellular carcinoma (HCC) is the most prevalent form of primary liver cancer, accounting for 75–85% of all liver cancer cases worldwide [1]. It remains a major contributor to the global cancer burden, ranking as the third most common cause of cancer-related death [1]. Liver transplantation and surgical resection are the most effective curative interventions, providing long-term survival, especially for early-stage HCC. Liver transplantation offers superior survival and lower recurrence in patients with underlying cirrhosis, while surgical resection is appropriate for patients with preserved liver function [2].

Despite the benefits, only a minority of patients, often cited as 10–20%, are eligible for surgical resection at diagnosis, mainly due to late-stage presentation [3]. For patients who are not surgical candidates, ablative therapies have become a well-established, minimally invasive, curative-intent therapy. Ablation may be performed percutaneously under image guidance or intra-operatively. The percutaneous approach will be the focus of this review. Radiofrequency ablation (RFA) and microwave ablation (MWA) are the most widely adopted modalities, supported as first-line curative treatments for very early-stage HCC (Barcelona Clinic Liver Cancer (BCLC) 0, less than 2 cm) and as alternatives to resection in early-stage disease when surgery is not feasible, according to major guidelines including the BCLC, National Comprehensive Cancer Network (NCCN), and American Association for the Study of Liver Diseases (AASLD) [4,5,6]. More recently, robust data have also supported the use of radiation segmentectomy (RADSEG) transarterial radioembolization (TARE) as an ablative therapy, which is increasingly being investigated as a curative-intent embolotherapy for HCC [7,8,9]. Cryoablation has shown emerging promise but remains less established, while irreversible electroporation (IRE) and ethanol injection are niche approaches reserved for select tumors in challenging anatomic locations. Histotripsy is an investigational nonthermal technique with early-phase data.

This review aims to summarize the recent evidence and advances in ablative modalities in the management of HCC, with a focus on their role in curative-intent treatment, patient selection, technical considerations, and comparative outcomes with other therapeutic options. The reader should gain further understanding of appropriate patient selection for ablative modalities in HCC.

## 2. Thermal Ablation Techniques

### 2.1. Radiofrequency Ablation (RFA)

**Figure 1:**
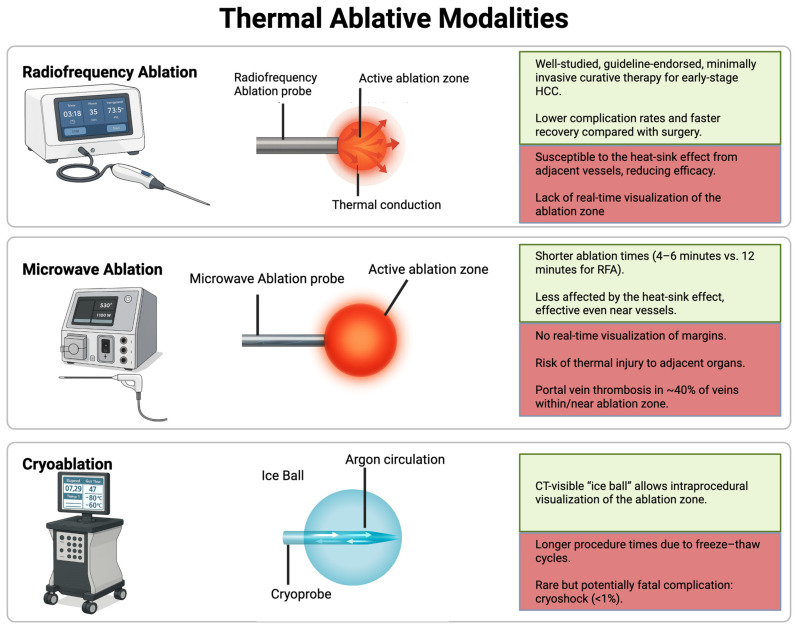
Thermal Ablative Modalities (schematic). Schematic overview of radiofrequency ablation, microwave ablation, and cryoablation. Green boxes summarize key advantages, while red boxes highlight principal limitations.

Since its adoption in the late 1990s, RFA has become one of the most extensively studied and widely accepted local therapies for early-stage HCC, particularly in patients who are not candidates for liver transplantation or surgical resection [10,11]. RFA utilizes alternating radiofrequency currents to generate ionic agitation and frictional heating, which drives water out of tissues, leading to coagulative necrosis [12]. While thermal injury to tumor cells begins at temperatures above 42 °C [13], temperatures must exceed 60 °C for effective ablation of hepatic tissue [12,13]. At this threshold, intracellular proteins denature and cell membranes are disrupted, resulting in immediate cell death and coagulative necrosis [12]. The heat-sink effect refers to the dissipation of thermal energy by adjacent blood flow in large vessels, which can reduce local temperatures and compromise treatment outcomes. Longer ablation times may be required for tumors with larger diameters when using RFA for tumors adjacent to a heat-sink (e.g., intrahepatic inferior vena cava) [14]. For hepatic tumors, ~5–15 min is typically required to achieve an ablation in tumors ≤ 3 cm and ~20–30 min in tumors > 3 cm, varying with tissue perfusion, probe type, and energy-delivery mode [15,16]. An overview of thermal ablation techniques, including RFA, is presented in Figure 1.

**Figure 2:**
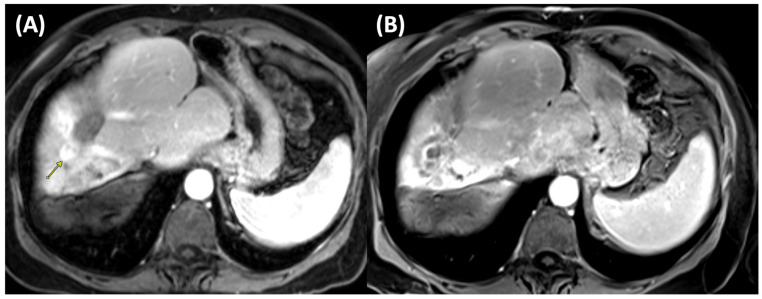
Pre- and post-treatment MRI after radiofrequency ablation (RFA). (**A**) Pre-treatment MRI shows a 1.4 cm arterially enhancing right hepatic tumor with washout, consistent with hepatocellular carcinoma (arrow). (**B**) Post-treatment MRI demonstrates no residual arterial enhancement. A thin rim of peripheral T1 hyperintensity surrounds the ablation cavity, representing expected post-procedural change.

RFA is generally well-tolerated and associated with low rates of significant complications. It has demonstrated satisfactory long-term survival outcomes when used as a first- or second-line therapy for HCC lesions measuring ≤3 cm [17]. Figure 2 illustrates pre- and post-ablation imaging findings of a patient undergoing RFA.

Achieving complete pathologic necrosis (CPN) defined as no viable tumor cells on tumor explant is considered an important endpoint for ablation therapy. The clinical significance of achieving CPN is highlighted by a landmark series of 501 liver transplant recipients treated with different locoregional therapies; patients with CPN had substantially lower recurrence rates (2.4% vs. 15.2%) and superior 5-year recurrence-free survival (73% vs. 56%) and disease-specific survival (99% vs. 86%) compared with those without CPN [18].

In The United Network for Organ Sharing (UNOS) database, thermal ablation (RFA/MWA) achieved CPN in 35.8% of ≤3 cm lesions [19]. A single-center RFA series reported CPN rates of 81.2% for tumors <2 cm, 63.6% for 2–3 cm, and 33.3% for >3 cm; perivascular tumors had lower CPN, attributed to heat-sink [20]. In a randomized control trial (RCT), 5-year overall survival was lower with RFA than resection (54.78% vs. 75.65%, *p* = 0.001), though RFA had fewer complications and shorter recovery [21]. The SURF trial, however, demonstrated no significant difference in 5-year overall survival (74.6% for surgery vs. 70.4% for RFA) or recurrence-free survival (42.9% vs. 42.7%), suggesting that surgery was not superior to RFA for small HCC, with more serious adverse events observed in the surgical group [22]. Nationwide registry data (*n* = 11,298) showed a 2.9% complication rate and 0.06% procedure-related mortality. Major complications are rare but include hemorrhage, visceral injury, tumor seeding, and skin burns. Post-ablation syndrome can be seen in up to 32% of patients. It typically consists of low-grade fever, delayed pain, nausea, vomiting, malaise, and myalgia, and is self-limiting, usually resolving within two weeks of the procedure [23].

Recent technological innovations in RFA for HCC have focused on improving the precision, safety, and efficacy, particularly for small tumors. New multi-electrode, high-power, incremental RFA systems, such as those using dual RF generators and separable clustered electrodes, allow for more controlled and complete ablation, resulting in lower local tumor progression and recurrence rates [24,25]. Prospective studies demonstrate that these systems significantly enhance targeting accuracy and safety [24,25]. RFA combined with immune checkpoint inhibitors (ICIs) has been explored to leverage RFA-induced immunogenic cell death to potentiate systemic antitumor immunity. In a prospective controlled trial, adding the anti-PD-1 agent toripalimab to RFA for recurrent HCC extended median recurrence-free survival to 15.4 months versus 8.2 months with RFA alone (HR 0.44; *p* = 0.04), with higher recurrence-free rates at 6, 12, and 18 months. Safety profiles were similar, with no significant rise in adverse events [26]. A propensity-matched analysis reinforced these results, showing 1-year recurrence-free survival rates of 32.5% for RFA + anti-PD-1 versus 10.0% for RFA alone (*p* = 0.001) and improved overall survival. Grade ≥3 immune-related adverse events were infrequent and occurred in 12.8% of combination-treated patients but were manageable [27].

Despite its broad utility, RFA has several limitations. RFA is limited by its relatively small ablation zone (typically up to 3 cm), often requiring 20–30 min and/or multiple applicators or overlapping applications to achieve sufficient margins, with efficacy attenuated by heat-sink from adjacent vessels, which can be technically challenging and less cost-effective [15,16,28]. Another important limitation is the lack of real-time visualization of the ablation zone, making it challenging to ensure complete tumor coverage during the procedure. Tumors that are poorly visualized on ultrasound or located near critical structures, such as the colon, diaphragm, or bile ducts, may be technically infeasible to treat safely [28]. However, adjunct techniques like artificial ascites or biliary cooling can help in some cases [28].

### 2.2. Microwave Ablation (MWA)

**Figure 3:**
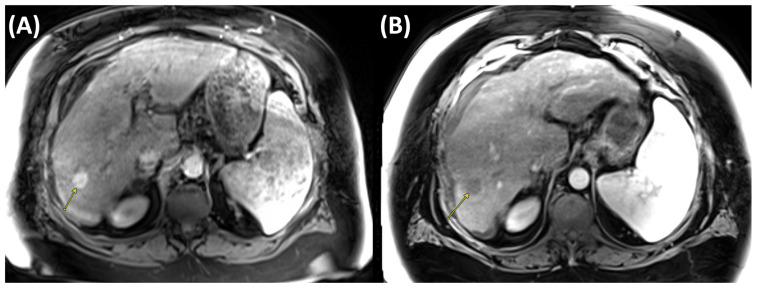
Axial contrast-enhanced MRI of the liver in the arterial phase demonstrating hepatocellular carcinoma (HCC) before and after microwave ablation (MWA). (**A**) Pre-treatment scan showing a hyperenhancing lesion in the right hepatic lobe measuring 2.0 cm (arrow). (**B**) Post-ablation scan demonstrating no arterial enhancement within the ablated tumor (arrow).

MWA operates primarily through the application of electromagnetic waves, which induce oscillation of water molecules within the tumor tissue. This oscillation generates heat, leading to localized hyperthermia and resulting in cellular necrosis and apoptosis of malignancies [29]. In order to effectively damage cellular structures and vascular integrity, this mechanism requires relatively high temperatures ranging from 60 °C to 100 °C [30]. Figure 3 illustrates pre- and post-ablation imaging findings of a patient undergoing MWA.

MWA offers several advantages over RFA. It achieves higher intratumoral temperatures, enabling larger ablation zones, and is particularly useful for more extensive tumors or those in challenging locations. The ability to deploy multiple antennas simultaneously expands treatment options and avoids the need for grounding pads typically required in RFA [29,31]. Treatment times are generally shorter with MWA, often under ten minutes per tumor [29], although this gap has narrowed with modern high-power RFA systems. Recent propensity-matched and single-center series with newer generators continue to show shorter ablation times for MWA, averaging 6–7 min compared with 9–10 min for RFA [31,32]. In a randomized trial, ablation time per lesion was 4–6 min for MWA versus 12 min for RFA, yet mean procedure duration remained similar (81 min [SD 13] for MWA vs. 84 min [SD 11] for RFA; *p* = 0.37) [33]. This reflects that ablation constitutes only a small fraction of overall procedure time, which is more strongly influenced by anesthesia and needle positioning. Another important advantage of MWA is its relative resistance to the heat sink effect, maintaining effective thermal delivery even when abutting blood vessels [29].

A compelling body of evidence from prior studies demonstrates that MWA achieves outcomes comparable to those of RFA and surgical resection. Numerous RCTs have demonstrated non-inferiority in local progression rates, indicating MWA’s effectiveness in preventing tumor recurrence at similar rates to those achieved with RFA [34]. In HCC tumors 1–3 cm, histopathology-confirmed CPN rates after MWA are ~77.8%, with literature-reported ranges from 57–95% depending on tumor size, number, and vessel proximity [35]. In a study by Xu et al., including 142 patients with 294 nodules within the “up-to-seven” criteria, percutaneous MWA achieved 1-, 3-, and 5-year overall survival rates of 97.2%, 75.4%, and 50.6%, respectively, with a median survival of 43 months and major complications in 2.8% (median tumor size up to 7 cm) [36]. MWA is generally well tolerated, with major complication rates of 2–5% and procedure-related mortality <0.1% at high-volume centers [37]. Complications include hemorrhage, bile duct injury, hepatic abscess, tract seeding, and thermal injury to adjacent viscera. These complications are observed across all thermal ablation techniques, not exclusively MWA. To reduce these risks, adjunctive methods like hydrodissection and the no-touch technique are employed. Hydrodissection involves instilling fluid (commonly 5% dextrose or saline with dilute contrast) into the perihepatic or perivisceral space to separate vulnerable structures (e.g., bowel, diaphragm, gallbladder) from the ablation zone, creating a protective barrier and enabling complete ablation in challenging locations [38]. The no-touch technique avoids direct puncture of the tumor by placing applicators circumferentially in surrounding liver tissue, ablating from the periphery inward. This reduces seeding risk and enhances margin control, though it requires multiple applicators and a larger treated liver volume [39].

Recent advances in MWA for HCC have centered on procedural optimization and combination strategies. Newer platforms, such as the Emprint™ microwave ablation system (Medtronic, Minneapolis, MN, USA), employ Thermosphere™ technology to stabilize the microwave field and heat distribution, resulting in more predictable, spherical, and uniform ablation zones [40]. This addresses limitations of conventional MWA, which can yield variable ablation geometries, particularly in challenging anatomical locations or larger tumors [40]. Compared with RFA, MWA with this technology has shown lower metastatic recurrence and HCC-specific mortality in tumors >2 cm, single nodular disease, or AFP-L3-negative HCC [40].

Despite the advantages of MWA, there are essential considerations regarding its limitations. A key limitation, similar to RFA, is the absence of real-time visualization of the actual ablation zone, which makes it difficult to confirm adequate margins during treatment. In addition, the risk of thermal injury to adjacent organs and vessels persists, particularly when tumors are near sensitive structures such as the bowel, diaphragm, or major vasculature [41,42]. The higher temperatures can inadvertently lead to collateral damage, resulting in complications such as perforation, bleeding, or thrombosis [29,41]. Portal vein occlusion occurs in nearly 40% of veins within or abutting the ablation zone, particularly in vessels <3 mm, reflecting the high susceptibility of small-caliber veins to thermal injury. This rate is markedly higher than for hepatic arteries (14%). Importantly, portal vein thrombosis did not correlate with increased local tumor progression, in contrast to patent hepatic arteries within the ablation zone, which were significantly associated with recurrence which could due to the heat-sink effect preserving microvascular tumor invasion [43]. Careful patient selection and pre-procedural imaging assessments are crucial to minimize such risks.

### 2.3. Cryoablation

**Figure 4:**
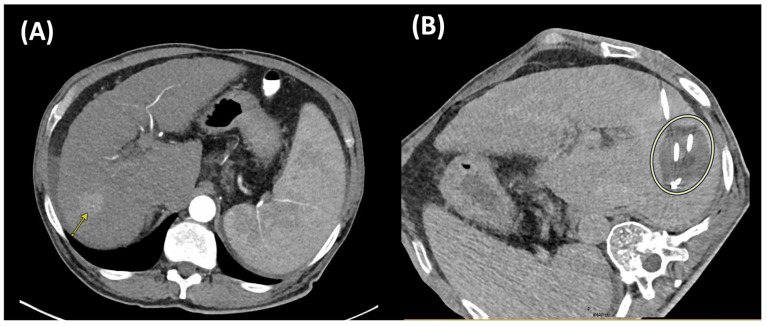
Axial CT imaging before and during cryoablation. (**A**) Pre-treatment scan demonstrating a 2.7 cm arterially enhancing HCC in the right lobe (arrow). (**B**) Intraprocedural CT during cryoablation with four applicators, obtained during the second freeze cycle, showing the characteristic ice ball measuring 4.5 × 4.0 cm (yellow circle).

Cryoablation is a minimally invasive ablative modality that achieves tumor necrosis via intracellular ice crystal formation, osmotic disturbances, and occlusion of small vessels. These biological processes ultimately lead to hypoxia, ischemia, and tissue death [44]. Current cryoablation approaches utilize the Joule–Thomson effect [45], i.e., a significant temperature decrease due to rapid expansion of pressurized gases through a narrow aperture, resulting in extreme cold temperatures, i.e., −20 to −40 °C, causing freezing and destruction of targeted tumor tissue [46]. A freeze–thaw-freeze protocol is typically used to induce tumor cell death [47]. The standard regimen consists of two freezing cycles, each lasting approximately 8 to 10 min, separated by a thawing phase of 5 to 8 min. This results in a total ablation time of approximately 20–30 min, which is longer than that of RFA and MWA. To ensure an adequate margin of treatment, the critical lethal isotherm of −20 °C is usually extended 0.3 to 0.5 cm beyond the tumor boundary [48]. A cryoablation case is illustrated in Figure 4, with imaging before and after treatment.

Cryoablation’s effectiveness is supported by visualization of the ablation zone and the CT-visible “ice ball.” The ice ball corresponds to tissue cooled to ≤0 °C, whereas the true ablation zone (lethal zone) is defined by the −20 °C isotherm (and in some tissues as low as −40 °C), which lies within the ice ball. Consequently, the visible ice ball consistently overestimates the true ablation zone. Intraprocedural visualization is typically achieved via computed tomography (CT) or ultrasound. This is particularly beneficial when targeting tumors adjacent to critical structures, as it helps minimize the risk of nontarget thermal injury [48]. Depending on the size and shape of the tumor, cryoablation may involve the use of multiple cryoprobes or the application of overlapping ablations to ensure complete coverage [49]. Compared to other thermal ablative modalities (i.e., MWA and RFA), cryoablation offers a more distinct ablative margin, a larger treatment zone when multiple applicators are used, and is associated with reduced periprocedural pain, as well as lower rates of vascular and biliary complications [50].

Historically, heat-based ablation modalities such as RFA and MWA have been the mainstay of local therapy for patients with HCC. More recently, cryoablation has emerged as an alternative, with growing but still limited comparative data supporting its role. Chunping et al. demonstrated that cryoablation and RFA had comparable efficacy and safety, with similar 5-year overall survival rates for cryoablation and RFA (1-, 3-, and 5-year OS: 97% vs. 97%, 67% vs. 66%, and 40% vs. 38%, respectively; *p* = 0.747). Cryoablation, however, showed improved local tumor control for lesions >3 cm, with 3-year local tumor progression rates of 7% versus 11% for RFA (*p* = 0.043). Complication rates were similar between groups, both below 4% [51,52]. Another comparative study suggested that cryoablation achieved better local tumor control in HCC lesions exceeding 2 cm compared with both RFA and MWA [52]. A meta-analysis comparing cryoablation with MWA reported no significant differences in complete ablation, recurrence, or survival outcomes, though cryoablation was associated with a lower major complication rate [53]. These findings suggest a potential role for cryoablation among other ablative therapies, though its comparative benefits remain to be validated in larger prospective studies.

Cryoablation is theorized to preserve tumor antigens by inducing cell death through freezing, which leads to the release of intact tumor-associated antigens. This process can enhance antitumor immunity by promoting dendritic cell activation and subsequent T-cell priming, potentially resulting in both local and systemic immune responses [54,55]. This results in converting immunologically “cold” tumors into “hot” tumors, characterized by increased cytotoxic T-cell infiltration and a more pro-inflammatory tumor microenvironment [56,57]. However, the immune response generated by cryoablation alone could be insufficient to achieve optimal tumor control due to the immunosuppressive tumor microenvironment and upregulation of immune checkpoints. Combining cryoablation with immune checkpoint inhibitors (ICIs), such as anti-PD-1 and anti-CTLA-4 antibodies, has shown to inhibit the growth of untreated tumors, promote tumor cell apoptosis, and prolong the survival time in preclinical HCC models [58,59]. These strategies are under active investigation with the goal of improving long-term outcomes and reducing recurrence in HCC patients undergoing cryoablation.

Limitations of cryoablation include longer procedure times compared to other ablative techniques due to multiple freeze/thaw cycles and the lack of effective intraprocedural hemostasis due to minimal coagulative effect. A rare but potentially life-threatening complication known as cryoshock has been reported in less than 1% of liver cases [60]. Cryoshock is characterized by a systemic inflammatory response syndrome (SIRS) leading to multiorgan failure. Clinically, patients present with rapid-onset hypotension, high fever, coagulopathy, renal failure, disseminated intravascular coagulation, and progressive organ dysfunction within hours to days after ablation. While its exact pathogenesis remains unclear, it is hypothesized to result from the abrupt release of intracellular contents, pro-inflammatory cytokines, and necrotic debris into the systemic circulation following large-volume hepatic tumor ablation. The liver’s rich vascular and immunologic environment may amplify this systemic response, particularly in cases involving extensive tumor burden or large ablation zones. Risk factors proposed in the literature include the number and size of lesions treated, the presence of underlying cirrhosis, and pre-existing systemic inflammation [60]. Historical concerns over cryoshock and the risk of hepatic fracture have limited the widespread adoption of cryoablation in hepatic applications, making it less commonly used than RFA or MWA.

### 2.4. Thermal Ablation Combined with Transarterial Chemoembolization

For tumors greater than 3 cm, ablation alone is associated with higher recurrence and lower overall survival compared to smaller lesions. This has driven research into combination strategies, particularly the integration of transarterial chemoembolization (TACE) with thermal ablation modalities [61,62].

RCTs and meta-analyses demonstrate that RFA combined with TACE significantly improves outcomes compared to RFA alone. For tumors up to 7 cm, TACE-RFA reduces the risk of death by 45% and the risk of recurrence by 34% [61,62]. These improvements are attributed to TACE mitigating the heat sink effect and addressing micrometastases, thereby enabling more complete and durable ablation [61,62]. MWA has also been evaluated in combination with TACE, with strong emerging evidence of benefit. In a 2022 prospective study of medium-sized HCC (3–5 cm), TACE-MWA achieved significantly higher objective response (95.2% vs. 61.9%, *p* = 0.02) and disease control rates (95.2% vs. 66.7%, *p* = 0.045) compared to TACE alone [63]. Median time to progression was prolonged nearly threefold (19.8 vs. 6.8 months, *p* < 0.001), and overall survival was higher at one and two years (100% and 95% vs. 95% and 76%, *p* = 0.032), without increased major complications [63].

Meta-analytic data from 14 studies including 1477 patients reinforced these findings, showing that TACE-MWA yielded the highest odds of long-term overall survival (OR 4.81, 95% CI 1.44–16.08, *p* = 0.011) and objective response (OR 3.93, 95% CI 2.34–6.61, *p* < 0.001), with maximal benefit in patients <60 years and tumors ≤3 cm [64]. Complication rates were favorable compared to MWA monotherapy [63,64]. Combination therapy with cryoablation and TACE also demonstrates superior outcomes compared to TACE alone, particularly for unresectable or large tumors. In very large tumors (≥10 cm), median overall survival was significantly longer with TACE-cryoablation (11.0 vs. 6.0 months for 10–15 cm tumors, *p* = 0.008; 8.0 vs. 5.0 months for ≥15 cm tumors, *p* = 0.001) [65]. The adverse event profile was favorable, with most complications mild to moderate (CTCAE grade 1–2) and grade 3–4 events in <4% of patients [65,66]. Mortality was not increased compared to TACE alone [66]. When comparing the three combination strategies, a recent meta-analysis of 42 studies including 5468 patients (TACE + RFA: 3398; TACE + MWA: 1477; TACE + cryoablation: 593) found that TACE-MWA provided the most effective, particularly in younger patients (<60 years) with tumors ≤3 cm. TACE-MWA demonstrated the highest odds of long-term overall survival (OR 4.81, 95% CI 1.44–16.08, *p* = 0.011) and objective response (OR 3.93, 95% CI 2.34–6.61, *p* < 0.001) compared to TACE-RFA and TACE-cryoablation. Recurrence-free survival at one year was similar between TACE-MWA (OR 4.61, 95% CI 1.70–12.51, *p* = 0.003) and TACE-RFA (OR 5.21, 95% CI 2.13–12.75, *p* < 0.001). Disease control rates were greater with TACE-MWA (OR 4.01, 95% CI 2.66–6.04, *p* < 0.001) and TACE-cryoablation (OR 4.05, 95% CI 1.68–9.74, *p* = 0.002) than with TACE-RFA (OR 3.23, 95% CI 2.14–4.86, *p* < 0.001.

Combined thermal ablative therapies (RFA, MWA, or cryoablation) with TACE demonstrate superior survival, recurrence, and tumor control outcomes versus monotherapy. The optimal combination strategy is likely to be patient- and tumor-specific, influenced by tumor burden, technical feasibility, and institutional expertise.

## 3. Non-Thermal Ablation Techniques

### 3.1. Irreversible Electroporation (IRE)

**Figure 5:**
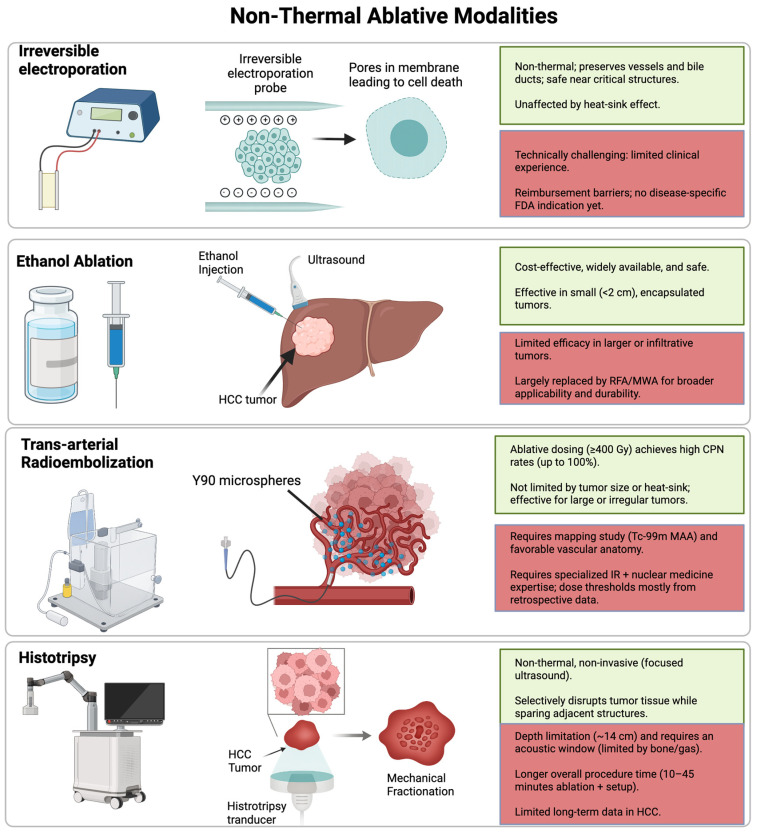
Non-Thermal Ablative Modalities (schematic). Overview of trans-arterial radioembolization, irreversible electroporation, histotripsy, and percutaneous ethanol injection. Green boxes summarize advantages, while red boxes note limitations.

Irreversible electroporation (IRE) is a novel, non-thermal form of ablation that relies on the delivery of short, high-voltage electrical fields via probes that induce microscopic holes in tumor cell membranes [67]. During this apoptotic process, the surrounding extracellular matrix, as well as nearby blood vessels and bile ducts are preserved within the liver [68]. IRE is usually performed via a percutaneous approach guided by ultrasound or CT. It is advantageous in cases where thermal ablation is contraindicated due to proximity to critical structures and is unaffected by heat-sink effects [48,69]. Robotic-assisted percutaneous IRE has also been demonstrated as an application for HCC treatment, resulting in enhanced precision [70]. Key non-thermal ablation techniques, including cryoablation, are summarized in Figure 5.

Multiple studies have demonstrated safety and efficacy of IRE as an ablative modality for HCC [71,72], with good complete response rates and long-term local recurrence-free survival [73], even when compared to RFA [74]. Comparable outcomes have specifically been reported for HCC lesions smaller than 3 cm when treated with IRE versus thermal ablation techniques [75,76]. Additionally, studies have shown that IRE can be safely employed in treating patients with tumors adjacent to intrahepatic bile ducts and may offer better tolerability compared to MWA [77,78]. A meta-analysis involving 776 patients with malignant liver tumors, of whom 285 (36.7%) had HCC, demonstrated favorable pooled survival outcomes following IRE. The pooled overall OS at 24 months was 61.5 (95% CI: 52.81–69.46, I^2^ = 0%), and the pooled PFS at 24 months was 49.0% (95% CI: 11.47–87.73, I^2^ = 96%). Importantly, the presence of HCC was associated with improved OS at both 12 and 36 months compared with non-HCC liver malignancies. While the overall complication rate was 23.7%, major complications (classified as grade C to F based on the Society of Interventional Radiology guidelines) occurred in only 6.9% of patients [79,80]. A 2023 multicenter study in Sweden assessed the outcomes of IRE for the treatment of 206 hepatic lesions (HCC and colorectal cancer liver metastases (CRCLM)) in 149 patients. IRE demonstrated a favorable safety profile, with low morbidity and satisfactory efficacy, particularly for patients with liver tumors situated in locations unsuitable for conventional thermal ablation techniques. The treatment appeared more effective in managing HCC compared to CRCLM [81]. Preliminary results from the first RCT comparing IRE and RFA in 33 patients with small HCC suggest that both approaches are effective, with no end point observed for PFS or OS and a 100% complete response rate based on imaging in both groups [82]. An emerging, though still uncommon, application of IRE is as a bridging therapy prior to liver transplantation, a strategy previously established for RFA and MWA [83]. In select cases where tumors are in close proximity to major hepatic vessels and other ablative modalities may be less suitable, IRE has been explored as an alternative. A 2019 case series reported patients with HCC lesions located near major hepatic vessels. All patients underwent IRE as a pre-transplant intervention. At 12 months, overall survival was 80%, with one patient death due to transplant-related complications. Pathological examination of the explanted livers revealed no evidence of vascular invasion [84]. Furthermore, five out of six (83%) IRE-treated HCCs showed CPN, while the sixth had less than 5% viable cells at the periphery, providing rare histologic confirmation of IRE’s ablative efficacy [84]. These findings underscore the potential of IRE as a nonthermal bridging strategy, particularly for tumors in anatomically challenging locations. Nonetheless, larger studies with explant correlation are warranted to validate these early results.

Recent technological advances have further refined IRE’s role in HCC management. High-frequency IRE (H-FIRE) modifies the standard delivery of unipolar pulses by using high-frequency bipolar bursts typically centered at 250 to 500 kHz with voltages of 1500 V/cm [85]. This approach produces a more homogeneous electric field, raises the threshold for nerve and muscle stimulation, and can eliminate all visible or tactile muscle contractions even at the highest tested energy bursts [85]. Preclinical studies in rat brain tissue demonstrated that 180 bursts with a pulse on time of 200 µs produced only a 3.5 °C temperature rise near the electrodes, with a 0.3% probability of thermal cell death, confirming preservation of the nonthermal mechanism [86]. Clinically, a phase 1 trial in 100 patients with localized prostate cancer treated with H-FIRE reported no intraoperative complications, a 6% rate of clinically significant recurrence at 6 months compared with approximately 20% in historical controls [87,88]. Probe placement, a well-recognized technical challenge in IRE given the need for parallel alignment within millimeters, has also been improved with robotic needle placement systems such as the CE marked and FDA cleared Epione^®^ platform (Quantum Surgical, Montpellier, France). In comparative studies, robotic assistance reduced the time from planning CT to ablation start from 87.4 min to 63.5 min (*p* < 0.001), lowered the radiation dose length product from 4714 to 2132 mGy·cm (*p* < 0.001), and improved mean probe placement accuracy from 3.1 mm to 2.2 mm (*p* < 0.001) [89]. In a bicentric prospective study of 21 patients with HCC or liver metastases, robotic-assisted IRE was feasible in 95.7% of lesions, with no adverse events reported and successful treatment achieved in all but one case [90]. However, these findings remain preliminary, and more data are needed in HCC, though they provide important guidance for future developments.

Limitations of IRE include the technical challenge of placing multiple electrodes in parallel with minimal divergence [69]. IRE ablations can also be influenced by interactions between electrical fields and variability in tissue impedance. Development of bipolar IRE probes is currently in progress and may help address these technical challenges [48]. Although the Food and Drug Administration (FDA) approved IRE for the ablation of soft tissues in 2008, it does not currently hold a disease-specific indication, which may hinder reimbursement and clinical practice adoption [91].

### 3.2. Percutaneous Ethanol Injection (PEI)

Ethanol (ETOH) ablation is a percutaneous chemical ablation technique in which dehydrated alcohol (99% ethanol, Ablysinol^®^ [BPI Labs, LLC, Largo, FL, USA]) is injected directly into the tumor, producing coagulative necrosis through cellular dehydration, protein denaturation, and small vessel thrombosis [80]. It is most effective for small HCC measuring less than 2 cm, where the risk of microsatellite disease is low (<20%) and most lesions are encapsulated, limiting the need for wide margins [92]. These tumor characteristics allow ETOH ablation to reliably achieve an adequate tumor response while preserving surrounding parenchyma.

Histopathologic outcomes support its efficacy. Shiina et al. reported CPN in 13 of 18 cases (72%) following PEI, with higher rates when combined with transcatheter arterial embolization [93]. Lin et al. demonstrated imaging based complete response rates of 88% with conventional PEI and 92% using higher-dose PEI in a RCT of HCC ≤ 4 cm [94]. These findings highlight that technique optimization and appropriate patient selection can meaningfully improve histologic outcomes. Its precision makes it particularly valuable for tumors adjacent to central bile ducts, bowel, or other critical structures, where thermal techniques like RFA or MWA may risk collateral injury [95]. The ablative effect is largely confined within the tumor, making it advantageous in preserving surrounding anatomy.

The procedure is performed under ultrasound or CT guidance, with a narrow-gauge single needle or multiport needle [95]. Treatment is delivered either in staged low-volume sessions (10 mL/session under local anesthesia) or, less commonly, as a “one-shot” injection of 30–50 mL under general anesthesia for larger tumors [80]. Multiple sessions (≥2) are often required to achieve complete coverage. While RFA has shown superior recurrence-free survival in larger lesions [96], ethanol ablation remains a cost-effective, widely available, and well-tolerated therapy, with a lower tract seeding risk than RFA [97]. Abdominal pain is common (up to 48%) but typically self-limited [98]. Ethanol ablation has since been largely replaced by more advanced ablative techniques such as RFA, and MWA, which offer broader applicability and improved local control in larger or less accessible tumors. However, in settings where cost is prohibitive or RFA/MWA technology is not feasible, ethanol ablation continues to serve as an effective, low-cost, and well-tolerated treatment for small HCC.

## 4. Radiation-Based Ablative Approaches

### 4.1. Trans-Arterial Radioembolization

**Figure 6:**
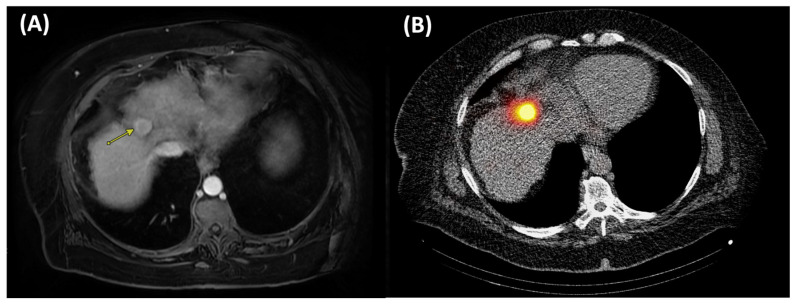
Pre- and post-treatment imaging of hepatocellular carcinoma (HCC) in a challenging location for percutaneous ablation due to the location near the heart and diaphragm. This patient was treated with Yttrium-90 transarterial radioembolization (Y90 TARE) RADSEG with ablative intent. (**A**) Axial post-contrast T1-weighted MRI before treatment shows a 2.7 cm hyperenhancing liver lesion (arrow). (**B**) Axial SPECT/CT after Y90 TARE demonstrates bremsstrahlung radiation from Y90 microspheres concentrated within the tumor. The patient underwent liver transplantation 4 months later, with complete pathologic necrosis on histologic examination of the liver explant.

TARE is an intra-arterial brachytherapy that uses yttrium-90-loaded microspheres infused via the hepatic artery to deliver high-dose beta radiation selectively to tumors while sparing surrounding parenchyma [99]. Two main microsphere types are available: glass microspheres (TheraSphere^®^, Boston Scientific) with high specific activity and minimal embolic effect that allows high-dose delivery without stasis [100], and resin microspheres (SIR Spheres^®^, Sirtex Medical) with lower specific activity that require a greater particle load and produce a more embolic effect [101]. Both devices are FDA-approved for local tumor control of unresectable solitary HCC (1–8 cm) in Child–Pugh A patients with well-compensated liver function, no macrovascular invasion, and good performance status [102]. It is endorsed in major guidelines for BCLC stages A, B, and C, where surgery, ablation, and transplantation are not feasible [103]. TARE can also be implemented for neoadjuvant downstaging, intermediate stage HCC not amenable to TACE, and advanced cases with portal vein tumor thrombosis (PVTT) where other embolic therapies carry high ischemic risk [104,105,106,107]. Unlike thermal ablation, TARE is nonthermal and unaffected by the heat sink effect, enabling treatment of tumors regardless of size, geometry, or proximity to heat sink vessels [100]. Figure 6 illustrates pre- and post-treatment imaging findings from a patient treated with TARE.

In recent years, TARE has undergone a paradigm shift from being primarily a palliative option for patients ineligible for surgery or ablation to a potentially curative-intent ablative therapy when delivered with segmental or subsegmental ablative dosing to small sectors of tumor-bearing liver [108]. This evolution is best exemplified by RADSEG, a high-dose, selective TARE approach first described by Riaz et al. [109] and now supported by robust retrospective and prospective evidence. RADSEG involves super selective catheterization of up to two Couinaud segments to deliver very high absorbed doses. Retrospective analyses have identified minimum absorbed dose thresholds for consistently achieving CPN as 400 Gy with glass microspheres and 433 Gy with resin microspheres, although prospective validation remains necessary [110,111,112]. For example, the LEGACY study demonstrated that patients with early–stage HCC treated with RADSEG at >400 Gy had an objective response rate of 89.8%, 100% local control by mRECIST, and CPN in all transplanted explants [110]. Similarly, the prospective RASER trial delivered median absorbed doses of 584 Gy, achieving a 100% objective response, durable complete responses in 90% of patients, and CPN in all transplanted livers [8] Both LEGACY and RASER were performed using glass microspheres with a growing literature on RADSEG with resin microspheres.

TARE has been compared with TACE, thermal ablation, and surgical resection. In a large transplant cohort directly comparing histopathologic outcomes after segmental or subsegmental therapy, TARE achieved a markedly higher CPN rate than TACE (83.3% vs. 29.0%, *p* < 0.0001), despite treating tumors of similar mean size (2.4 cm for TARE vs. 2.3 cm for TACE). The median delivered TARE dose was 603.8 Gy. After propensity score matching to adjust for baseline differences, TARE maintained superior CPN rates (80.9% vs. 31.9%) while requiring fewer treatments per lesion, underscoring its greater ablative efficacy in the pretransplant setting [113]. The comparative efficacy of RADSEG and percutaneous ablation has also been explored in several matched cohort analyses. Biederman et al. [114] compared TACE plus MWA (MWA; *n* = 80) with RADSEG (*n* = 41) in treatment-naïve patients with solitary HCC ≤ 3 cm. After propensity score matching, complete response rates, time to progression, and OS were similar. In a subsequent matched cohort of 68 patients with solitary HCC ≤ 4 cm, RADSEG and MWA had comparable toxicity, objective response (91% vs. 83%; *p* = 0.55), OS (59.0 mo vs. 44.3 mo; *p* = 0.20), and non-target liver progression-free survival, but RADSEG achieved significantly longer target tumor PFS (57.8 mo vs. 38.6 mo; *p* = 0.005), likely due to improved margin control with cone-beam CT guidance. Furthermore, RADSEG has demonstrated oncologic equivalence to surgical resection in select patients. Kim et al. [115] compared TARE (*n* = 57) with surgical resection (*n* = 500) in patients with HCC ≥ 5 cm. Overall survival (OS), time to progression, and intrahepatic progression were similar (OS HR, 0.98; 95% CI, 0.40–2.43; *p* = 0.97; time to progression HR, 1.10; 95% CI, 0.55–2.20; *p* = 0.80; intrahepatic progression HR, 1.45; 95% CI, 0.72–2.93; *p* = 0.30). TARE was associated with fewer complications and shorter hospital stays, supporting a favorable safety profile. Notably, the TARE cohort included patients with local vascular invasion. In a multicenter study by De la Garza-Ramos et al. [116], the authors found no OS difference between RADSEG and resection for solitary HCC ≤ 8 cm, with similar progression rates (33% vs. 32%). These results firmly position RADSEG as an intra-arterial transcatheter modality that warrants consideration in addition to resection or thermal ablation in select early-stage patients.

TARE is not constrained by tumor size in the same way as thermal ablation and can achieve excellent local control for lesions >3 cm when delivered selectively at ablative doses [8,110,111,114,117,118,119]. However, data regarding the impact of tumor size on achieving CPN remain somewhat conflicting. In the LEGACY trial explant analysis, baseline tumor size was not associated with CPN (*p* = 0.35). By contrast, Sarwar et al. reported that tumors achieving CPN were smaller, with a median diameter of 2.2 cm (IQR, 1.8–3.0 cm), compared to 3.4 cm (IQR, 2.8–4.8 cm) for tumors with incomplete necrosis (*p* = 0.03). These findings suggest that while TARE can be effective across a wide tumor size range, outcomes may still favor smaller lesions, a point that remains to be explored further. TARE also allows for ablation of irregularly shaped tumors or multifocal disease within a targeted arterial territory. Its minimal embolic effect makes it safe for patients with PVTT, where other locoregional therapies risk ischemic liver injury [120,121,122,123]. Successful ablative TARE requires favorable vascular anatomy for selective delivery, adequate hepatic reserve, the ability to achieve high tumor doses without exceeding safety thresholds for the uninvolved liver, and the use of cone beam CT for tumor confirmation and margin assessment [100,124,125].

Despite its advantages, TARE has several important limitations. The procedure typically requires a separate mapping day with technetium-99m macroaggregated albumin (Tc-99m MAA) imaging to assess hepatopulmonary shunting and determine dosing [124]. Lung shunt fraction >30 Gy/session or >50 Gy cumulative is a contraindication due to the risk of radiation pneumonitis [125]. However, this rarely occurs with the RADSEG approach. Success depends on favorable vascular anatomy for selective catheterization. Variant anatomy or arteriovenous shunting may preclude optimal delivery [100]. The procedure requires significant interventional radiology expertise and institutional support from nuclear medicine and radiation safety. Moreover, current ablative dose thresholds, such as the 400 Gy benchmark, remain derived primarily from retrospective analyses in small tumors and require validation in larger prospective studies [110,111,112].

### 4.2. External Beam Radiotherapy

Radiation therapy for HCC can also be delivered as external beam radiotherapy (EBRT) rather than TARE. EBRT refers to the noninvasive delivery of high-energy radiation beams or charged particles from outside the body, directed at the liver using advanced image guidance [126]. Radiation exposure to the normal hepatic parenchyma has been a historical concern with EBRT; however, advances in stereotactic body radiotherapy (SBRT) can now deliver high, conformal doses to focal liver tumors with lower dosing to the uninvolved parenchyma [127]. In an expert consensus paper, ablative EBRT was defined as treatment delivering a biologically effective dose (BED_10_) of at least 80–100 Gy [127]. Two forms of SBRT are currently used. Photon-based SBRT delivers radiation dose continuously along the beam path, while proton-based SBRT exploits the Bragg peak to deposit the majority of energy at a defined depth with minimal exit dose. Despite these mechanistic differences, both approaches have shown broadly similar effectiveness in HCC treatment [128]. In contrast to TARE, EBRT is not currently included in the 2022 BCLC guidelines as a standard treatment option [103]. Nevertheless, retrospective studies suggest encouraging outcomes when EBRT is applied with ablative intent. A recent multicenter retrospective analysis directly compared ablative EBRT and RADSEG as initial therapy for solitary HCC ≤ 8 cm [129]. Both modalities achieved high local control at one year (97% RADSEG vs. 93% EBRT), but RADSEG achieved faster and more complete radiologic response (97% vs. 82%; median time 1 vs. 7 months) and a higher rate of complete pathologic necrosis in transplanted explants (76% vs. 33%), though only 3 EBRT patients received transplant [129]. Overall survival and progression-free survival were similar, though EBRT was typically offered to older patients with larger tumors and poorer performance status [129]. Thus, while EBRT may serve as a valuable option in unique scenarios, such as patients with compromised hepatic arterial access (e.g., dissection or occlusion) or recurrence after prior locoregional therapies such as recent TACE, its role remains to be further defined. More rigorous comparative effectiveness studies are needed relative to catheter-based therapies like RADSEG for ablative intent.

## 5. Histotripsy

Histotripsy is a nonthermal ablation technique derived from the Greek words “histo” (soft tissue) and “tripsy” (to break). It leverages ultrasound pulses to mechanically disrupt tumor tissue [130,131]. A series of brief, focused, high-intensity ultrasound pulses lasting only microseconds is delivered to generate negative pressures that induce inertial cavitation without significant thermal effects [130,132,133]. Within the extracellular matrix, pre-existing nanoscale gas pockets expand into microbubbles when exposed to these ultrasound waves, exceeding the tissue’s tensile strength [134]. These bubbles can rapidly grow enormously before collapsing within microseconds, creating mechanical forces that fragment cells into subcellular debris. Multiple pulses are typically required to achieve complete ablation. Because cavitation is threshold-dependent, histotripsy selectively disrupts tumor tissue while sparing adjacent critical structures [135]. Moreover, these pulses can be tuned to specific tissue types depending on tumor location.

The THERESA Study was the first human clinical trial evaluating histotripsy for liver cancer [136]. It established the technique’s ability to effectively destroy tumor tissue while avoiding device-related adverse effects [137]. The study met its primary endpoint of safely generating focal ablation zones and secondary endpoint to identify device-related adverse effects, where transient elevations in transaminases were noted but returned to baseline within one week. Building on this progress, the HOPE4LIVER trial further evaluated the safety and efficacy of histotripsy in patients with primary and metastatic liver tumors [138]. Technical success was achieved in 95% of treated lesions within 36 h. Major procedure-related complications occurred in 6.8% of patients (3/44) [138]. These findings supported the FDA approval of histotripsy for liver cancer treatment in October 2023. At one-year follow-up, the trial demonstrated an overall survival rate of 73.3% in patients with HCC. The one-year local tumor control rate was 63.4% based on the primary endpoint and 90% based on a post hoc assessment [139]. A comparative summary of complete pathologic necrosis, survival outcomes, and key limitations across all ablative modalities is provided in Table 1.

Notable limitations of histotripsy include the depth limitation of 14 cm and obstruction of ultrasound delivery by gas or bone, limiting its use to certain anatomical locations. Because lesions must be clearly visible on ultrasound, tumors not detectable with this modality are not candidates for histotripsy. Hollow, gas-filled organs are particularly susceptible to damage [141], and acoustic energy attenuation reduces effectiveness in deeper tissues, posing challenges in obese patients [142]. Treatment duration is another important limitation. Although the actual ablation usually requires 10–45 min, the entire procedure may take longer due to positioning, imaging, and setup, which is longer than most established therapies such as RFA or MWA. Lesion size also remains a challenge since there are no standardized guidelines regarding the maximum treatable dimension [143]. In practice, tumors up to 4 cm have been effectively treated in a single session, whereas larger lesions require multiple overlapping treatment zones, an approach that prolongs procedure time and increases technical complexity [143]. Histotripsy may also induce vascular thrombosis, risking ischemic injury in highly vascular organs [144]. In a multicenter safety analysis of 230 patients (510 tumors; 31 HCC patients), portal vein thrombosis was reported in 6 cases (2.6%), highlighting this as a clinically relevant complication [145]. Although prior reports have suggested minimal risk of metastasis, concerns about potential tumor cell dissemination remain, given the mechanical disruption of tumor architecture inherent to cavitation-based therapy [146,147,148].

## 6. Imaging Advances in Ablation

A major advancement in percutaneous tumor ablation has been the use of navigation and fusion imaging to overcome limitations of conventional ultrasound guidance, particularly for small, isoechoic, or poorly visualized lesions [149]. Fusion imaging merges real-time ultrasound with pre-acquired CT, PET/CT, or MRI, markedly enhancing lesion conspicuity, procedural feasibility, and technical success across RFA, MWA, cryoablation, and IRE. In RFA, the incorporation of fusion imaging has led to measurable gains in technical success and local tumor control. Meta-analysis data show a 98.1% technical efficacy rate, with significant reductions in local tumor progression (LTP) risk (RR = 0.61, *p* < 0.001) and complication rates (RR = 0.70, *p* < 0.03) compared with US alone [149]. Prospective studies have confirmed improved tumor localization confidence and technical feasibility, with technical success rates up to 96.8% and 1-, 2-, and 3-year LTP rates of 8.6%, 12.2%, and 15.2%, respectively [150,151,152]. For lesions invisible on US, the use of fusion imaging and cone-beam CT has achieved technical success of 91–95% and 1-year LTP rates of only 0–4.5% [25,151]. MWA has similarly benefited from these technologies, with registry data showing technical success rates of 93.9% and low complication rates [153,154]. The addition of three-dimensional fusion imaging has further refined needle placement and margin assessment, achieving complete response rates of 100% and 1- and 2-year LTP rates of 7.0% and 9.4% [151]. In both RFA and MWA, confirmation of an ablative margin >5 mm through fusion imaging has been shown to reduce LTP by 16 percentage points [155,156,157]. Although cryoablation and IRE remain less commonly applied, recent reports demonstrate that navigation and FI facilitate accurate targeting in challenging anatomical locations or near critical structures [158]. Post-ablation margin assessment has also advanced with fusion imaging, enabling real-time confirmation and immediate supplementary ablation. This approach achieves technical success rates of 81–100% and technical effectiveness rates of 89–100% at first follow-up [159]. Intraprocedural CT-CT fusion has been shown to improve 2-year LTP-free survival in HCC from 74% to 97% (HR 0.21, *p* = 0.037) [157]. Three-dimensional contrast-enhanced ultrasound fusion has revealed that 26% of tumors deemed adequately treated by 2D imaging actually had margins <5 mm; only margins ≥5 mm were associated with a 0% 3-year LTP rate, compared with 20.7–27.2% for smaller margins [155]. In anatomically complex locations such as the caudate lobe, both tumor size >2 cm and lack of a ≥5 mm margin independently predict LTP [160]. Fusion imaging will play an increasingly important role in the future of percutaneous tumor ablation by enabling precise targeting and margin assessment across diverse clinical scenarios. Further high-quality studies are needed across all ablation modalities to optimize integration, standardize protocols, and validate long-term oncologic benefits.

## 7. Conclusions

Ablative techniques present an essential curative-intent option for HCC, with thermal, nonthermal, and emerging modalities enabling safe and effective treatment across diverse tumor scenarios. Each technique carries unique advantages and limitations, highlighting the need for individualized, multidisciplinary selection. Continued innovation and prospective comparative studies will refine the optimal integration of these therapies into personalized HCC care.

## Figures and Tables

**Table 1:** Comparison of Modalities for Hepatocellular Carcinoma: Complete Pathologic Necrosis, Overall Survival, and Limitations.

Modality *	Complete Pathologic Necrosis (CPN) **	Overall Survival (OS) **	Key Limitations
Radiofrequency ablation	61.6% overall in explant series; median tumor size 2.0 cm (range, 1.6–2.6) [20]	54.8% 5-year OS (vs. 75.6% for resection, RCT, *n* = 230) [21]	Heat-sink near vessels; smaller single-applicator zones; overlaps often needed; tumor visibility/adjacent structures can limit use
Microwave ablation	92.8% histology-confirmed with biopsies; mean tumor size 4.1 ± 1.9 cm (range, 1.2–8.0) [35]	50.6% 5-year OS (single-center prospective, *n* = 142, 294 nodules, median size ≤7 cm) [36]	Collateral thermal injury risk near critical structures; may require hydrodissection
Cryoablation	NR (imaging complete response 97% within Milan criteria cohort) [60]	40.0% 5year OS (vs. 38% RFA, comparative cohort, *n* = 360) [51]	Longer procedure times; minimal coagulative hemostasis; rare cryoshock <1%
Trans-arterial Radioembolization-Radiation Segmentectomy	100% CPN achieved when absorbed doses exceeded: 400 Gy with glass microspheres (median tumor size 2.5 cm, range 1.3–8.0). 433 Gy with resin microspheres (median tumor size 2.6 cm, range 1.9–3.4) [8,110,111,112]	75.0% 5-year OS for solitary tumors ≤3 cm and 57.0% for tumors ≤ 5 cm [9,108].	Requires mapping study (MAA); anatomy must permit selective delivery; lung-shunting may limit dosing/safety; early stasis (resin microspheres) can cap achievable dose; specialized expertise
Ethanol Ablation	72% CPN; tumor size range 1.6–5.0 cm (average, 2.9 cm) [93]	49.0% % 5-year OS (single center retrospective cohort, *n* = 685) [140].	Multiple sessions usually needed; Limited to small encapsuled tumors; tumor capsule limits any potential for ablation margin
Irreversible electroporation	83% CPN in explant series of transplanted patients; median tumor size 2.2 cm (range, 1.6–2.6) [84]	40.9% 3-year OS (meta-analysis, *n* = 776, 285 HCC) [79]	Requires GA with neuromuscular blockade; parallel multi-probe placement; tissue-impedance variability; reimbursement barriers
Histotripsy	NR	73.3% 1-year OS (HOPE4LIVER prospective trial, *n* = 44, liver tumors including 31 HCC) [139]	Tumor must be sonographically visible. Depth limit ~14 cm; requires clear acoustic window (blocked by gas or bone); efficacy reduced in deep lesions/obesity; possible vascular thrombosis; multi-target patient repositioning

Abbreviations: HCC, hepatocellular carcinoma; CPN, complete pathologic necrosis; OS, overall survival; GA, general anesthesia; MAA, macroaggregated albumin; NR, not reported. * Please refer to Ahmed et al. [80] for detailed definitions and standardized terminology ** The OS and CPN data are presented for summarization rather than direct comparison due to differences among included patients.

## Data Availability

No new data were created or analyzed in this study. Data sharing is not applicable to this article.

## Abbreviations

The following abbreviations are used in this manuscript:

BCLC	Barcelona Clinic Liver Cancer
CP	Child-Pugh
CPN	Complete pathological necrosis
CRCLM	Colorectal cancer liver metastases
ECOG	Eastern Cooperative Oncology Group
FDA	Food and Drug Administration
HCC	Hepatocellular carcinoma
mRECIST	Modified Response Evaluation Criteria in Solid Tumors
MWA	Microwave ablation
OS	Overall survival
PFS	Progression-free survival
PVTT	Portal vein tumor thrombosis
RCT	Randomized controlled trial
RFA	Radiofrequency ablation
RADSAG	Radiation segmentectomy
TACE	Transarterial chemoembolization
TARE	Transarterial radioembolization
